# Emerging mosquito-borne flaviviruses

**DOI:** 10.1128/mbio.02946-24

**Published:** 2024-10-31

**Authors:** Amy N. Nelson, Alexander Ploss

**Affiliations:** 1 Department of Molecular Biology, Princeton University, Princeton, New Jersey, USA; The Ohio State University, Columbus, Ohio, USA; University of Wisconsin-Madison, Madison, Wisconsin, USA

**Keywords:** flavivirus, infectious disease, emerging viruses, species tropism, antiviral immunity

## Abstract

Flaviviruses comprise a genus of enveloped, positive-sense, single-stranded RNA viruses typically transmitted between susceptible and permissive hosts by arthropod vectors. Established flavivirus threats include dengue viruses (DENV), yellow fever virus (YFV), Zika virus (ZIKV), and West Nile virus (WNV), which continue to cause over 400 million infections annually and are significant global health and economic burdens. Additionally, numerous closely related but largely understudied viruses circulate in animals and can conceivably emerge in human populations. Previous flaviviruses that were recognized to have this potential include ZIKV and WNV, which only became extensively studied after causing major outbreaks in humans. More than 50 species exist within the flavivirus genus, which can be further classified as mosquito-borne, tick-borne, insect-specific, or with no known vector. Historically, many of these flaviviruses originated in Africa and have mainly affected tropical and subtropical regions due to the ecological niche of mosquitoes. However, climate change, as well as vector and host migration, has contributed to geographical expansion, thereby posing a potential risk to global populations. For the purposes of this minireview, we focus on the mosquito-borne subgroup and highlight viruses that cause significant pathology or lethality in at least one animal species and/or have demonstrated an ability to infect humans. We discuss current knowledge of these viruses, existing animal models to study their pathogenesis, and potential future directions. Emerging viruses discussed include Usutu virus (USUV), Wesselsbron virus (WSLV), Spondweni virus (SPOV), Ilheus virus (ILHV), Rocio virus (ROCV), Murray Valley encephalitis virus (MVEV), and Alfuy virus (ALFV).

## INTRODUCTION

Phylogenetic analyses have concluded that mosquito-borne flaviviruses likely originated in Africa (1, 2). With extended dry seasons, mosquitoes sought out water sources and discovered reserves stored by humans. Thus, some mosquito species, in particular *Aedes aegypti*, invaded and infested human-populated regions and subsequently developed a biting preference for humans (3). The urban transmission cycle of mosquito-vectored viruses was thereby initiated, and global viral spread was facilitated by insect, animal, and human migration and international trade.

**Fig 1:**
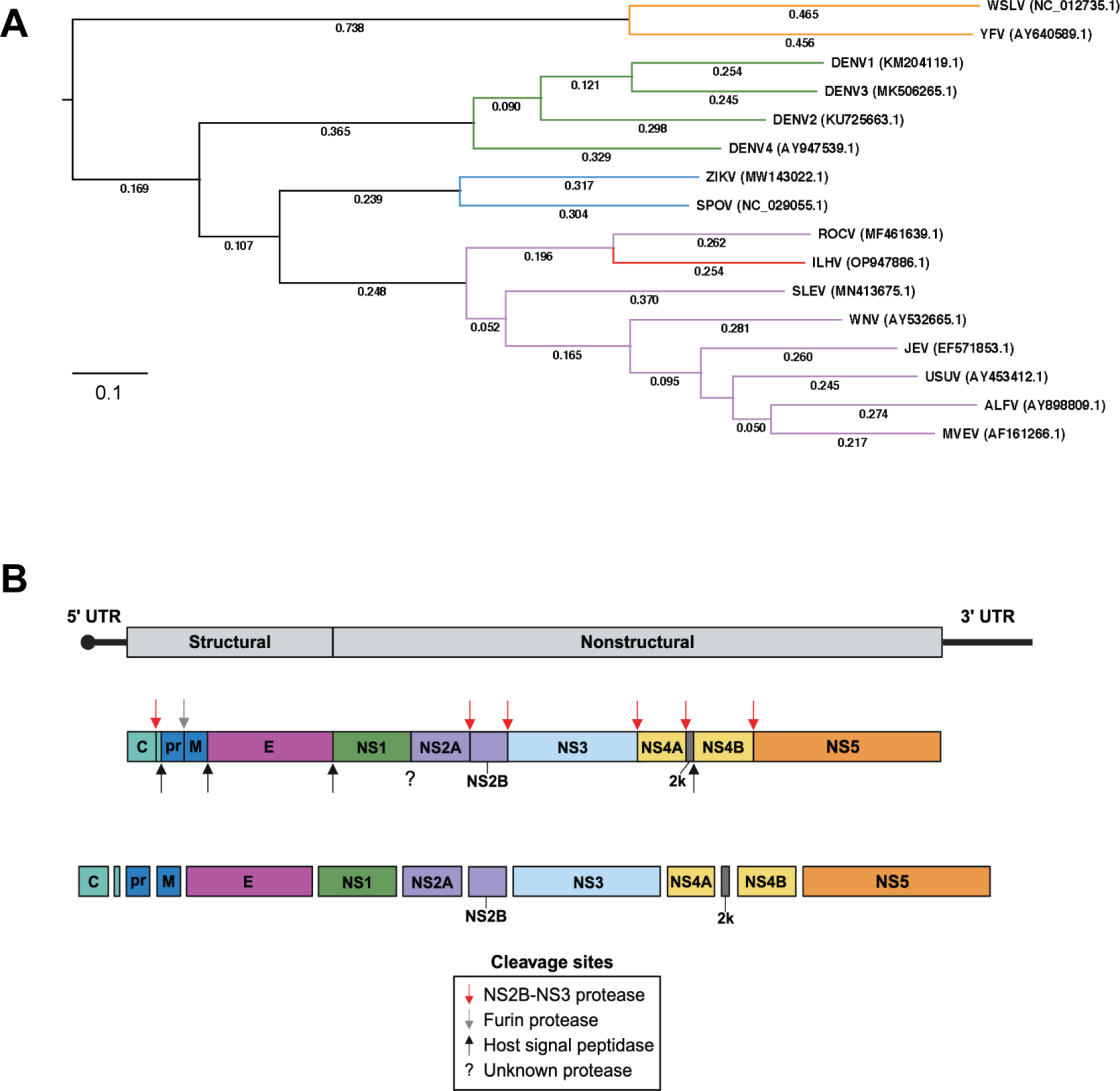
Phylogeny and genome structure of emerging mosquito-borne flaviviruses. (**A**) Phylogenetic tree of the emerging flaviviruses Wesselsbron virus (WSLV), Spondweni virus (SPOV), Rocio virus (ROCV), Ilheus virus (ILHV), Usutu virus (USUV), Alfuy virus (ALFV), and Murray Valley encephalitis virus (MVEV) with reference to established flavivirus threats including yellow fever virus (YFV), dengue virus serotypes 1–4 (DENV1-4), Zika virus (ZIKV), Saint Louis encephalitis virus (SLEV), West Nile virus (WNV), and Japanese encephalitis virus (JEV). Multiple sequence alignment of full genome sequences was performed using the MAFFT algorithm, and phylogenetic analysis was performed using the randomized accelerated maximum likelihood (RAxML) program with 100 bootstrapping iterations available from DNASTAR Lasergene MegAlign Pro (Madison, WI, United States). Serocomplexes are differentially colored with YFV in orange, DENV in green, SPOV in blue, JEV in purple, and Ntaya in red. The GenBank accession numbers of the viral strains used for the alignment are indicated in parentheses. (**B**) Flavivirus genome organization. Flavivirus genomes are approximately 11 kb long with 5' and 3' UTRs and a 5′ terminal cap structure. The coding region contains one ORF, which is translated as a single polyprotein. Host and viral proteases cleave the polyprotein into three structural proteins (C, prM, E) and seven non-structural proteins (NS1, NS2A, NS2B, NS3, NS4A, NS4B, and NS5). The host signal peptidase (black arrow) has cleavage activity in the endoplasmic reticulum lumen, while the viral NS2B-NS3 protease (red arrow) is active in the cytoplasm. In the Golgi apparatus, a furin protease (gray arrow) separates the pr and M proteins. Of note, the protease that cleaves between NS1 and NS2A is currently unknown.

Over 3,500 mosquito species exist among 41 genera, yet flavivirus transmission is accomplished by two main genera: *Aedes* and *Culex* (4). Historically, *Aedes*-associated flaviviruses (YFV, DENV, and ZIKV) have caused epidemics, and *Culex*-associated flaviviruses (WNV and JEV) have been responsible for significant outbreaks of encephalitic disease. In general, *Aedes*-associated viruses cause viscerotropic or hemorrhagic symptoms, while *Culex*-associated viruses cause neurotropic symptoms (5). A notable exception is ZIKV, which is *Aedes*-associated, yet neurotropic and unique as the only flavivirus capable of being sexually transmitted between humans (6–8). Emerging *Aedes*-associated flaviviruses include Spondweni virus (SPOV) and Wesselsbron virus (WSLV), while emerging *Culex*-associated flaviviruses include Usutu virus (USUV), Ilheus virus (ILHV), Rocio virus (ROCV), and Murray Valley encephalitis virus (MVEV). Established and emerging flaviviruses that share the same mosquito vector tend to be more closely related as demonstrated by phylogenetic analysis (Fig. 1A). As climate change progresses, the ecological niches of mosquitoes will continue to expand, thereby exposing new populations to vector-borne diseases. Additionally, viral adaptation in new hosts may contribute to the emergence of novel strains that could exhibit differences in cellular or host tropism.

## FLAVIVIRUS GENOME ORGANIZATION AND REPLICATION

In nature, mosquito-borne flavivirus transmission initiates when an infected mosquito bites its host. As the mosquito extracts a blood meal, it releases virus and saliva that is rich in anti-inflammatory and anti-coagulant proteins. It is hypothesized that the virus first interacts with skin-resident immune cells before migrating to draining lymph nodes and gaining widespread access to additional cell types and host tissues.

Despite their differences in virulence as well as cellular and host tropism, all flaviviruses share a similar overall genome structure. Their positive-sense single-stranded RNA genome is approximately 11 kb pairs long and encodes one open reading frame (ORF) that is translated as a single polypeptide at the endoplasmic reticulum (ER) membrane (Fig. 1B). Host and viral proteases co-translationally and post-translationally cleave the polypeptide into three structural proteins and seven non-structural (NS) proteins (9). Interestingly, the ER protease that cleaves the C-terminus of NS1 remains unknown (10). The structural genes encode the proteins capsid (C), membrane (M), and envelope (E). The nonstructural (NS) genes (1, 2A, 2B, 3, 4A, 4B, and 5) encode proteins that support viral replication and evasion of the host antiviral response. Untranslated regions (UTRs) are present at the 5′ and 3′ ends of the genome, which are approximately 100 bp and 400–600 bp long, respectively. They are highly structured and contain necessary elements for viral replication (11). Flavivirus genomes have type I cap structures (m7GpppAmG) at the 5′ end and lack poly(A) tails at the 3′ end.

The flavivirus replication cycle begins with attachment to host cells and receptor engagement, followed by internalization, endosomal trafficking, capsid disassembly, initial translation and genome replication, virion assembly and maturation, and release of viral progeny. As the sole surface glycoprotein, E interacts with attachment factors on the host cell membrane to increase affinity for virus particles and receptors, which permits internalization. The host cell receptor for E remains unknown and is an ongoing area of investigation. It is possible that multiple entry factors are at play and may vary by virus, but this gap in knowledge hinders efforts to develop effective therapeutics and vaccines. While the interplay between flaviviruses and the host cell surface is not fully understood, it has been well-established that virions are internalized via clathrin-mediated endocytosis. Within endosomes, they are trafficked to the ER and undergo a process of acidification. This causes a conformational change of the virion, which enables fusion between the viral and endosomal membranes. The nucleocapsid is subsequently released into the cytoplasm and disassembly liberates the viral genome. Initial translation occurs on host cell ribosomes. Threading of the polyprotein through the ER membrane induces a curvature that leads to formation of replication organelles (ROs) or vesicle packets (VPs). These invaginations provide a replication niche shielded from detection by host pattern recognition receptors in the cytoplasm. Within the ROs, the mature NS proteins assemble into the correct structure to serve as the replication machinery known as the replication complex (RC). A double-stranded RNA (dsRNA) intermediate is produced, and the negative-sense strand subsequently serves as the template for the production of nascent positive-sense genomes.

## THE EMERGENCE OF ZIKA VIRUS AND WEST NILE VIRUS AND HISTORICAL OUTBREAKS IN HUMAN POPULATIONS

Prior to causing epidemics, ZIKV and WNV were both considered emerging viral threats but were the focus of few studies. Expectedly, research of these viruses exponentially increased as they gained human relevance. ZIKV was incidentally discovered in 1947 during routine surveillance for YFV in mosquitoes and primates in Uganda and was named after the Zika forest (12). The main vectors of this virus are *Aedes aegypti* and *Aedes albopictus*. For decades, ZIKV circulation was restricted to equatorial African and Asian countries, infecting nonhuman primates (NHPs), and on rare occasions, humans causing mild febrile illness and skin rashes. In 2007, the first major ZIKV outbreak in humans occurred in the Federated States of Micronesia with 49 confirmed and 59 probable cases. This was the first record of ZIKV infection outside of Africa and Asia (13). The next emergence of ZIKV took place in French Polynesia from 2013 to 2014 with an estimated 32,000 symptomatic infections (14). The largest ZIKV epidemic occurred 2015–2016 in Brazil with an estimated 497,593 to 1,482,701 cases as reported by the Ministry of Health (15). This outbreak coincided with an upsurge of Guillain–Barré syndrome and babies born with microcephaly or other neurological defects. In 2016, the World Health Organization declared ZIKV as the source. Retrospective studies of clinical cases in French Polynesia also confirmed a causal relationship between ZIKV infections and increased incidence of Guillain–Barré syndrome in adults and microcephaly in newborn babies that were exposed during the first trimester (16, 17). Further, the first clinical case of ZIKV in the continental U.S. occurred in 2016, and local transmission was reported in Texas and Florida counties in 2017 (18–20). In recent years, ZIKV has been detected in Europe (21) and India (22), which demonstrates the significant capacity for flaviviruses to geographically expand (21, 22).

WNV was discovered in 1937 and isolated from a febrile patient in the West Nile district of Uganda (23). It is primarily vectored by *Culex pipiens* and utilizes wild birds, in particular crows, as amplifying hosts. Symptoms include headache and febrile illness; however, the majority of human cases are asymptomatic. During an epidemic in Israel in 1957, symptoms of neuroinvasive disease were observed for the first time in elderly patients. These symptoms were seen throughou11t sporadic outbreaks in Israel, Egypt, France, and South Africa in the following decades (24–27). Further, cases of meningoencephalitis were recorded during outbreaks in Romania and Russia in the late 1990s (28, 29). The 1999 epidemic in New York marked the emergence of WNV into the Western hemisphere and was accompanied by a striking incidence of encephalitis (30). Initial serological tests suggested that Saint Louis encephalitis virus (SLEV) was the source; however, concurrent cases of avian viral encephalitis and significant mortality in birds suggested otherwise. Although SLEV caused an outbreak of encephalitis in Missouri in 1933 with over 1,000 symptomatic cases and 200 deaths (31, 32), it was known to rarely cause death or disease in avian species (33, 34). Further testing and sequencing revealed WNV as the etiological agent (35, 36). WNV expanded across the United States, causing an estimated 7 million infections between 1999 and 2016 (37, 38). Cases continue to be reported each year and coincide with the warm weather season (39). In addition to causing neurotropic disease in humans, WNV has caused astounding lethality in bird species globally. American crows and blue jays have been particularly affected, and bird migration likely contributed to viral spread across North America.

## EMERGING FLAVIVIRUS THREATS

**Fig 2:**
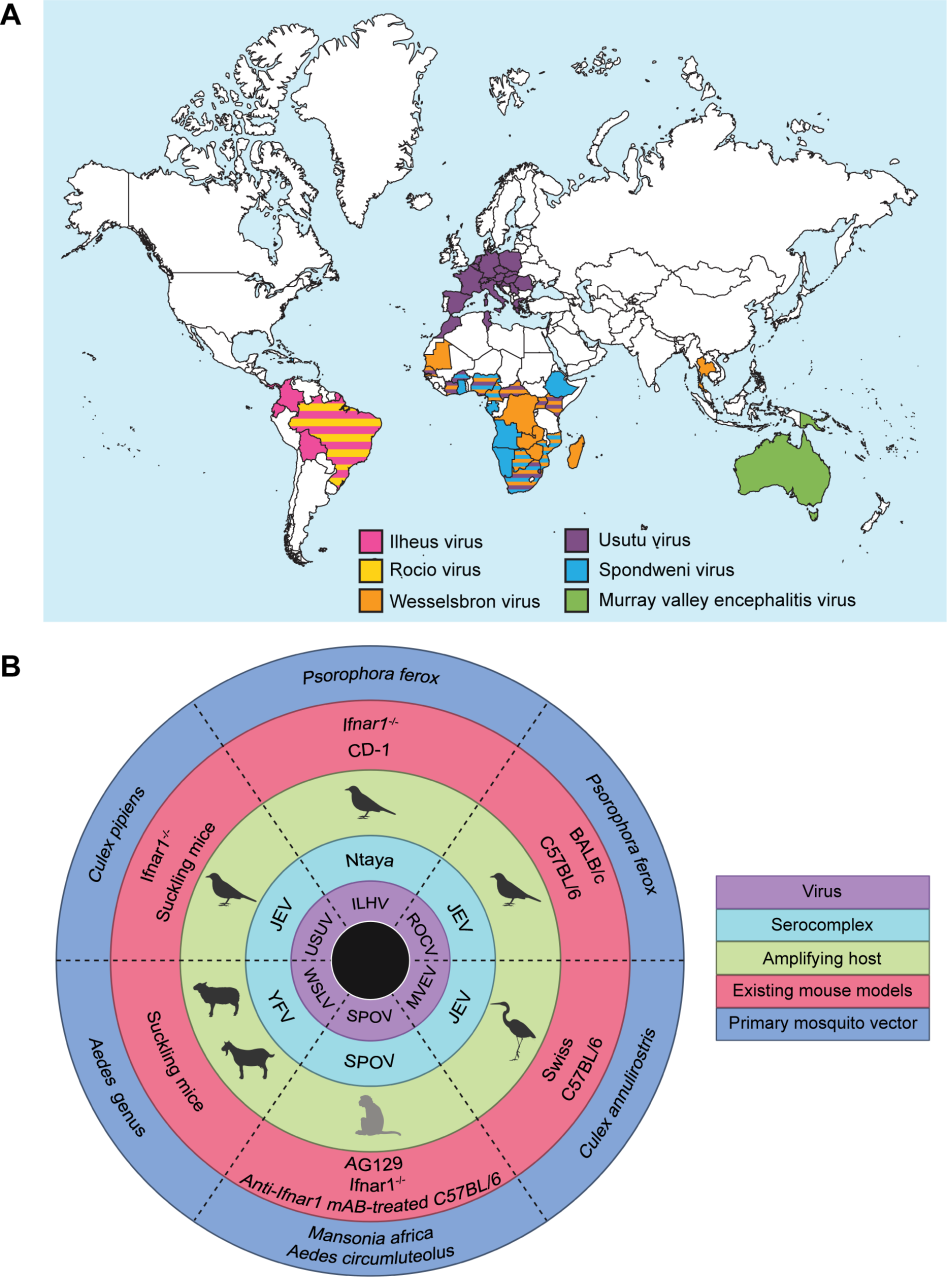
An overview of six emerging mosquito-borne viruses: Ilheus virus (ILHV), Rocio virus (ROCV), Wesselsbron virus (WSLV), Usutu virus (USUV), Spondweni virus (SPOV), and Murray Valley encephalitis virus (MVEV). (**A**) Global distribution of these emerging flaviviruses based on molecular or serological detection in humans, animals, or mosquitoes. (**B**) Host range of these emerging flaviviruses. The innermost ring indicates the emerging viral species. Moving outwards, the following rings indicate the serocomplex, amplifying host, existing mouse models for each virus, and the primary mosquito vector(s) responsible for transmission of each virus. While antibodies to these viruses may have been detected in a variety of animal species, only the main amplifying host(s), which manifests clinical symptoms during infection, was depicted. While the amplifying host for SPOV is not officially known, it is assumed that non-human primates (NHPs) constitute this population. NHPs are the amplifying host for ZIKV, the most closely related virus to SPOV, and rhesus macaques have been proven to be permissive after experimental inoculation (40).

Neglected zoonotic viruses are longstanding threats to human health and emerging flaviviruses have been detected globally (Fig. 2A). They are commonly found in the same tropical and sub-tropical regions as established flaviviruses due to overlap in mosquito vectors. Major reasons to study these viral species include their prevalence and lethality in animal reservoirs and the capacity to infect humans.

### Usutu virus

In 1959, USUV was discovered in South Africa during a large-scale study investigating viral species present in arthropod hosts (41). The first strain was isolated from a female *Culex neavei* mosquito near its eponymous river in Eswatini (41). USUV belongs to the JEV serocomplex and often co-circulates with WNV, which has a similar transmission cycle between birds and ornithophilic mosquitoes (42, 43). *Culex pipiens* serve as the primary vector, but USUV has also been detected in the *Aedes* and *Mansonia* genera (42, 44). These mosquitoes transmit USUV between avian amplifying reservoirs, such as Eurasian blackbirds and Great Gray owls. Systemic infection occurs in these bird species characterized by splenomegaly, hepatomegaly, and detection of USUV antigen-positive cells in the spleen, liver, brain, heart, pancreas, lung, kidney, intestine, gizzard, skeletal muscle, and bursa of Fabricius (45). The introduction of USUV into Europe was marked by the mass mortality of blackbirds in Austria in 2001 (46). Retrospective studies have since linked the true first emergence of USUV in Europe to birds in Tuscany, Italy, in 1996 (47). USUV is currently found in Africa and Europe, but only the European strains are epizootic and pathogenic in bird species (48, 49). A handful of symptomatic USUV infections have been recorded in humans with manifestations of fever, jaundice, and skin rash. In 2009, neurological symptoms presented in two immunocompromised patients in Italy, including a woman undergoing chemotherapy and a woman receiving a liver transplant (50, 51). Three additional cases of neuroinvasive disease were recorded in Croatia in 2013 (52). Antibodies against USUV have been detected in birds, bats, squirrels, wild boar, deer, and horses (45, 53–57). Due to this spread, Many European countries have instilled USUV surveillance programs with 10 countries monitoring USUV and WNV in mosquitoes. Additionally, some countries monitor wild and captive birds, boars, deer, cattle, and equids (58).

### Wesselsbron virus

WSLV was first isolated in 1955 from the brain of a decomposed lamb and the blood of a febrile man in Wesselsbron, South Africa (59, 60) and was linked to abortion in domestic sheep. Today, WSLV is endemic in Africa and affects regions of South Africa in particular. It belongs to the YFV serocomplex and is mainly transmitted by *Aedes* mosquitoes between ruminant amplifying hosts including sheep, goats, and cattle. WSLV is teratogenic in ruminants as it can cause spontaneous abortions, congenital malformations, and stillbirths (60, 61). Adult sheep and goats often have asymptomatic infections or mild fever, whereas newborn lambs and children have an increased vulnerability marked by higher rates of mortality (62, 63). WSLV-specific antibodies have been isolated from a variety of domesticated animals, including dogs, camels, ostriches, pigs, donkeys, horses, and domestic fowls (64). Due to similar clinical manifestations, such as spontaneous abortions in livestock, WSLV cases in sheep were often initially misdiagnosed as Rift valley fever virus (RVFV), an arbovirus in the *Bunyaviridae* family. In humans, WSLV is subclinical or mild in most cases, but may cause acute, influenza-like illness, including fever, headaches, myalgia, and arthralgia. WSLV is restricted to Africa with the exception of WSLV isolation from mosquitoes in Thailand in 1966; however, there were no confirmed cases in any animal hosts. Only 33 human cases of WSLV have been recorded to date, the majority of which were due to laboratory exposure. One case of neuroinvasive disease occurred after direct transmission of viral suspension into the eye of a laboratory technician (59, 60, 65, 66). In Senegal, serological surveillance has indicated the presence of WSLV since the 1970s. In 2013, two novel strains of WSLV were isolated from febrile patients who were co-infected with malaria or hepatitis E. Both individuals fully recovered within a few days following peak symptoms (67).

### Spondweni virus

During field collection of mosquitoes in Zululand, SPOV was isolated from *Mansonia uniformus* in 1955 (68). Later studies revealed that SPOV was detected in the blood of a febrile patient in Uganda 3 years prior, but was classified as ZIKV due to serological cross reactivity (69, 70). As the only two members of the SPOV serocomplex, SPOV and ZIKV are close relatives sharing 75% amino acid sequence identity (71). There is limited molecular and serological evidence confirming SPOV infection in different animal species, thus drawing conclusions about amplifying and incidental hosts is complicated. Attempts to isolate SPOV RNA or antibodies from wild birds and rodents in South Africa were unsuccessful, but antibodies were detected in a handful of cattle and sheep (72). The primary vector and preferred host remain unspecified, but the latter is likely NHPs, similar to ZIKV. SPOV has mainly been detected in field-caught *Mansonia Africana* and *Aedes circumluteolus* mosquitoes (27, 73). Current studies of vector competence have conflicting results. One group demonstrated that *A. aegypti*, but not *Culex quinquefasciatus*, transmit SPOV (74), while another group demonstrated low susceptibility and viral dissemination by *A. aegypti*, *A. albopictus*, and *C. quinquefasciatus* (71). Interestingly, SPOV was detected in field-caught *C. quinquefasciatus* in Haiti in 2016, which was the first record of SPOV outside of Africa (75). Unique from other mosquito-borne flaviviruses, the potential for sexual transmission of ZIKV and SPOV has been demonstrated (76). SPOV has not caused any human epidemics, and only six clinical cases have been described in the literature with symptoms including fever, headache, myalgia, and jaundice (69, 77). It is assumed that most infections are self-limiting and often go undiagnosed. Serological surveillance studies indicate SPOV infections in at least 10 countries in Sub-Saharan Africa (78–80).

### Ilheus and Rocio viruses

As illustrated above, many flaviviruses originated in Africa; however, ILHV and ROCV were first detected in Brazil and continue to circulate throughout South and Central America. ILHV belongs to the Ntaya virus serocomplex, while ROCV is a member of the JEV serocomplex. Both viruses are neurotropic with transmission cycles involving primarily *Psorophora ferox* mosquito vectors and avian hosts. These viruses are closely related and share amino acid identities of approximately 75% for nonstructural protein 1 (81) and 79% for envelope protein (82). During surveillance for YFV in 1944, ILHV was isolated from *Psorophora* and *Ochlerotatus* mosquitoes in Ilheus, Bahia, Brazil (83). Prior to this, SLEV was the only neurotropic flavivirus detected in South America (84). Sporadic human infections have been recorded with one lethal case in an elderly, encephalitic patient in 2017 (85). No epizootics have been recorded in any animal species, but ILHV-specific antibodies have been detected in birds, rodents, bats, monkeys, horses, water buffalo, tortoises, and sloths (86–88). In contrast, an epidemic of human encephalitis marked the emergence of ROCV, which was named after a neighborhood in Sao Paolo, Brazil. This outbreak occurred from 1975 to 1976, causing over 100 deaths and neurological sequelae in 20% of survivors (89). The first ROCV strain was obtained from central nervous system (CNS) tissues of a male who succumbed to lethal infection within five 5 days of illness onset. Notably, ROCV has not caused additional outbreaks in humans; however, two ROCV infections were detected during a DENV outbreak in Brazil from 2011 to 2013. Both patients experienced fever, myalgia, and arthralgia, but fully recovered (90). Serological surveys indicate ROCV infection has occurred in birds, rodents bats, horses, water buffalo, and marsupials (88, 91, 92).

### Murray Valley encephalitis and Alfuy viruses

In 1951, MVEV was first isolated from encephalitic patients in Australia (93) and is a major cause of arboviral infections in Australia and Papua New Guinea today (94). It belongs to the JEV serocomplex and is transmitted between water fowl, such as herons and egrets, by *Culex annulirostris* mosquitoes (95). Between 1 in 150 and 1 in 1,000 human cases are symptomatic, and the case fatality rate is 15%–30% (96). Symptomatic disease is characterized by fever, nausea, vomiting, rash, and confusion (97, 98) with serious neurological symptoms, including seizures in children, encephalitis, acute flaccid paralysis, and tremors (96). Interestingly, MVEV can also cause neurological disease in horses (99). Several MVEV epidemics have occurred in Australia with 45 cases in 1951, 59 cases in 1974, and 17 cases in 2011 (100). These outbreaks were preceded by heavy rainfall and regional flooding, which caused migration of infected water fowl seeking dry shelter (101, 102). Following the 1951 outbreak, serological testing in wild birds revealed that 12 out of 16 water bird species were previously infected with MVEV while only 7 out of 24 land bird species were previously infected (103). Surveillance of sentinel chickens has also shown increased seroconversion to MVEV after heavy rain seasons and therefore may serve as a useful warning for human outbreaks (104). Alfuy virus (ALFV) was previously recognized as a naturally attenuated subtype of MVEV sharing 83% amino acid identity (105). The first ALFV strain was isolated in 1966 from a pheasant in Queensland (106), and additional strains were isolated from *Culex* mosquitoes in 1999 (107). ALFV has not been associated with disease in humans nor other animals, but investigation of this virus in comparison to MVEV may provide insight into natural mechanisms of flavivirus attenuation. Motifs in the ALFV envelope protein have been associated with increased binding to glycosaminoglycans (GAGs), a known mechanism of flavivirus attenuation (107).

## EXPERIMENTAL MOUSE MODELS FOR MOSQUITO-BORNE FLAVIVIRUS RESEARCH

### Applications and limitations of cell culture models

Molecular virology studies in cell lines are a low-cost platform that allows a high degree of experimental intervention. Determining the susceptibility and permissivity of different cell lines to specific viruses suggests tissue and organismal tropisms that can be further characterized by *in vivo* studies. Additional cell culture experiments include viral propagation and titration, characterization of replication kinetics, generation of reporter cell lines, and screening for therapeutic candidates. While *in vitro* studies provide crucial preliminary insights into the molecular mechanisms of a virus in a particular cellular environment, they are unable to model how viral pathogenesis transpires in an organism with a complex immune system. Animal models provide a means for studying viral pathogenesis to determine clinical manifestation and ability to cause viremia, tissue pathology, and lethality. In addition, novel viral strains are isolated from naturally infected mosquitoes and animals. Pathological findings in animals infected in the wild have confirmed the neurotropic capacity of some viruses.

### Commonly utilized mouse models

Historically, flavivirus research has been hindered by the scarcity of small animal models. Since wild-type (WT) mice are often resistant to flaviviruses, immunocompromised mice are commonly used. Some models have compromised adaptive immunity lacking functional B and T cells, while others have compromised innate immunity due to dysfunctional IFN signaling. Mice deficient in type I IFN (α, β) and/or type II IFN (γ) receptors, such as *Ifnar1*^−/−^ and AG129, have been particularly useful for flavivirus research. Suckling mice, which have underdeveloped immune systems, have demonstrated a susceptibility to some flaviviruses. The emerging threats discussed in this review have been studied in many of the aforementioned mouse models and are summarized below (Fig. 2B).

### USUV mouse models

Adult wild-type mice are not susceptible to USUV-related disease; however, *Ifnar1*^−/−^ mice, which lack a type I IFN receptor subunit, provide a useful model for studying USUV pathogenesis and neurovirulence. The median survival time of *Ifnar1*^−/−^ mice was 6 days post-infection (dpi) (108). These mice also served as a model to study the differential pathogenesis of African and European USUV clinical isolates: South Africa 1959, Senegal 2003, Spain 2009, Uganda 2010, and Netherlands 2016. For the former four strains, all *Ifnar1*^−/−^ mice succumbed to lethal infection by 6 dpi and viral RNA was detected in serum, spleen, liver, heart, and brain. In contrast, mice infected with the Netherlands 2016 strain had an 88% survival rate with less inflammation and lower viral burden in tissues. Peak viremia levels were highest in mice exposed to African strains, suggesting greater pathogenesis compared with the European strains. Additionally, it was concluded that Spain 2009 and Netherlands 2016 strains were likely the result of distinct introduction events of USUV into Europe (109).

One-week-old suckling mice currently serve as the only immunocompetent mouse model to study USUV pathogenesis and neurotropism. Dose-dependent survival was reported in 1-week-old Swiss mice as 84% of animals survived following inoculation with 1e2 PFU of USUV, while only 40% of animals survived after inoculation with 1e4 PFU. This study also investigated cross-protection between USUV and WNV in 1-week-old mice. Prior infection with USUV did not prevent infection following WNV challenge, yet it did protect against disease and death (110). Another study in 1-week-old swiss mice identified massive inflammation and high levels of viral RNA in the brain, spinal cord, eye, and sciatic nerve on 6 dpi, corresponding to a 60% mortality rate. Viral RNA was also found in the liver, spleen, kidney, hindlimb muscle, and bladder (111). Regarding USUV neurotropism, neuronal apoptosis and demyelination of white matter was observed in 1-week-old NMRI mice 7–9 dpi; however, 2-week-old mice were resistant to peripheral and neuroinvasive USUV infection (112).

### WSLV mouse models

African wild rodents were not susceptible to WSLV as no viremia nor symptom manifestation occurred (113). Suckling mice were found to be susceptible to WSLV by intraperitoneal (IP), intracerebral (IC), or subcutaneous (SC) injection. Peak viremia occurred 3 dpi *via* IC route. At this time, *A. aegypti*, but not *C. quinquefasciatus*, mosquitoes were able to transmit WSLV from infected to naïve suckling mice (114). Another study demonstrated an age-dependent susceptibility to WSLV. While adult mice successfully cleared WSLV, newborn mice succumbed to lethal infection. Infection of peritoneal macrophages isolated from mice of both ages recapitulated the *in vivo* findings. Electron microscopy suggested that macrophages from adult mice, but not newborn mice, phagocytosed WSLV particles (115). In 2013, WSLV infection in a black rat was recorded for the first time and isolated from brain tissue. This strain along with two human clinical isolates from the same region were passaged in suckling mice to obtain live virus. Extraction and sequencing of the genomic RNA determined that all three isolates were the same WSLV strain (67).

### SPOV mouse models

Based on previous knowledge of ZIKV transmission, the potential for sexual transmission of SPOV was studied in IFNα/β/γ receptor knockout (AG129) mice. The mean survival time after SPOV injection was 10.8 days. Tissue tropism was similar to ZIKV with comparable viral titers found in serum, brain, testes, epididymitis, seminal vesicles, and eyes; however, infectious SPOV was only detected in 4% of ejaculates compared to over 60% in mice infected with ZIKV strains. Further, infectious SPOV was only detected in ejaculates collected on day 10 post-infection, whereas infectious ZIKV was detected in ejaculates during a range from days 6–14, 5–12, or 5–23 post-infection depending on the inoculation strain (76). While the potential for sexual transmission of SPOV was described in this mouse study, it is evident that the potential for sexual transmission of ZIKV is much greater. Additional studies are required to understand why ZIKV has a higher capacity for sexual transmission, but one contributing factor could be the overall higher virulence of ZIKV compared with SPOV.

Another group studied the tropism of SPOV strains Chuku 1952 and South Africa 1955 in C57BL/6 mice. Inoculation with either strain resulted in no clinical symptoms; however, administration of an IFNAR1 blocking monoclonal antibody (mAb) in C57BL/6 mice prior to SPOV exposure led to lethal or persistent infection with high levels of SPOV RNA detected in the serum, spleen, kidney, and brain at 7, 14, and 21 dpi (116). An increase in IFNγ-producing CD8^+^ T cells was observed 8 dpi with no change in the frequency of CD4^+^ T cells. This group also investigated SPOV pathogenesis in 3-week-old human STAT2 knock-in (hSTAT2 KI) mice, which was established as an immunocompetent mouse model for ZIKV (117). While SPOV NS5 was found to bind hSTAT2, it did not promote its degradation, and viral replication was not supported in these mice. Notably, passive transfer of neutralizing DENV and ZIKV mAbs protected mice treated with the IFNAR1 mAb against lethal infection and weight loss following SPOV challenge, thereby demonstrating cross-reactivity among these flaviviruses (116). In pregnant mice, SPOV was observed to cause fetal demise in *Ifnar1*^−/−^ mice but not in IFNAR1 mAb-treated WT mice. SPOV RNA was detected in the placenta of both mice, but no overt fetal pathology was observed in mAb-treated WT mice. In contrast, moderate segmental necrosis of the brain and spinal cord and mild pulmonary inflammation were observed in pregnant *Ifnar1*^−/−^ mice (74, 116).

### ILHV and ROCV mouse models

Recently, pathogenicity of non-adapted and mouse-adapted ILHV strains was evaluated in CD-1 mice. In 4-week-old mice, intraperitoneal injection of a mouse-adapted strain caused 100% mortality with an average survival time of 5.6 days, while the non-adapted strain caused only 10% mortality. In 8-week-old mice, IP inoculation of the mouse-adapted strain caused 60% mortality, while SC inoculation caused 40% mortality. Unlike the mouse-adapted strain, the non-adapted strain was highly neurotropic with significant levels of virus detected in the brain, spinal cord, and eyes 4 dpi. Both ILHV strains caused 100% mortality in *Ifnar1*^−/−^ mice by 3 dpi (118).

Four-week-old mice BALB/c mice have been established as a model to study ROCV-induced meningoencephalomyelitis. ROCV was detected in the CNS 2 h post-infection (hpi) using a high dose, and 100% mortality occurred by 9 dpi. Infiltration of lymphomononuclear cells including CD8^+^ T cells and F4/80^+^ macrophages, neuronal degeneration, and high expression of caspase-3 was observed in brain tissue during late infection (119). Production of chemokine CCL2 was found to be required for macrophage infiltration into the brain. Interactions between CCL2 and its receptor CCR2 facilitated monocyte migration into the brain and Ccr2^−/−^ mice were found to have increased disease severity and mortality (120).

The ability of prior exposure to ILHV or SLEV to protect against lethal ROCV challenge has been studied in C57BL/6 mice. Nonlethal doses of ILHV or SLEV were administered at days 0 and 21 followed by lethal ROCV challenge on day 42. Prior infection with ILHV or SLEV conferred 100% or 70% protection against ROCV challenge, respectively (82).

### MVEV and ALFV

The neuroinvasive capacity of MVEV was first demonstrated in a study utilizing Swiss mice, which determined that encephalitis was an age-dependent symptom as younger mice had an increased vulnerability (121). Further, the neurotropism of this virus was confirmed by detection in the CNS following intravenous (IV) injection of MVEV in C57BL/6 mice. In adult C57BL/6 mice, 1e8 PFU of MVEV caused 100% mortality, while 1e4-1e5 PFU caused approximately 50% mortality via intravenous route. By intracranial injection, 1e2 PFU resulted in 100% mortality (122). Separately, two strains of MVEV, which differed by one amino acid in the E glycoprotein, exhibited drastically different degrees of neuroinvasion in 3-week-old Swiss mice. The highly neuroinvasive strain was first detected in lymph nodes 24 hpi and caused 100% mortality, while the lowly neuroinvasive strain generally caused subclinical infection and was not detected in lymph nodes. While both strains entered the CNS via the olfactory lobe, the lowly neuroinvasive strain remained restricted to this region and the forebrain, while the highly neuroinvasive strain became diffusely distributed (123, 124). Further investigation revealed that a neutrophil inflammatory response accompanied CNS invasion starting five dpi and is triggered by production of the proinflammatory cytokine TNF-α and neutrophil chemoattractant N51/KC (125). Additionally, BALB/c mice were protected from lethal challenge with the highly neuroinvasive strain by previous inoculation with MVEV subviral particles (126).

While ALFV is very closely related to MVEV and JEV serocomplex members are typically neurotropic, ALFV did not invade the CNS of 3-week-old Swiss mice after peripheral inoculation (105). In contrast, 71% of 6-week-old *Ifnar1*^−/−^ mice developed encephalitis following ALFV exposure. Of note, the average onset of mortality following ALFV infection was 12.1 days, which was significantly later compared to the 100% mortality observed 6 dpi with MVEV (105, 127). In comparison to MVEV, a novel attenuation marker of ALFV was discovered as a single amino acid change in domain III of the E protein, which conferred enhanced binding to GAGs and reduced neuroinvasion in 3-week-old Swiss mice (107).

## ADDITIONAL ANIMAL MODELS FOR MOSQUITO-BORNE FLAVIVIRUS RESEARCH

### Birds

Many bird species can serve as amplifying hosts for USUV, which necessitates an avian pathogenesis model. Juvenile chickens (1-day-old) are worthy candidates as they become viremic and shed virus detectable by oral and cloacal swabs (128). Further, heart tissue pathology aligned with myocarditis observed in USUV-infected wild birds (129). Domestic chickens have been used to study MVEV, SLEV, and WNV (130–132). Unfortunately, 2-week-old domestic geese and chickens were resistant to USUV infection (133, 134). In general, passerine birds are known to be susceptible to USUV (135, 136). Experimental inoculation of canaries resulted in 3 out of 10 animals developing viremia with brain lesions, immune cell infiltrates in the lung, splenomegaly, and pallor of liver observed at necropsy (137). In contrast, WNV was previously found to cause 100% lethality in domestic canaries, thus indicating that USUV is less pathogenic in this species (138).

In Brazil, house sparrows occupy the ROCV epidemic zone, which led investigators to evaluate their responses to experimental inoculation. Following infection, house sparrows became transiently and lowly viremic. As relatively inefficient hosts, they likely play a very minor role in ROCV transmission. Interestingly, ROCV-immune birds remained susceptible to SLEV challenge, whereas SLEV immunity protected birds against detectable viremia after ROCV challenge (139). In another study, young chicks (1–2 days old) became viremic for 4 days after ROCV exposure. Mosquitoes were allowed to feed on these chicks and two *Culex* species were able to transmit ROCV for the subsequent 20 days (140).

Few *in vivo* studies of MVEV have been reported, but low-dose experimental inoculation in little egrets, intermediate egrets, and pacific herons caused viremia. The onset of viremia occurred earlier in intermediate egrets compared with herons, which were viremic 1–4 dpi, but no difference in susceptibility was observed. Younger birds (≤2.5 months) had higher maximum viremia compared with older birds (≥ 8 months).

### Sheep

Early WSLV studies in newborn lambs recorded mortality in 10 out of 37 animals. Biphasic fever, anorexia, staring coats, increased respiratory rate, lethargy, and general weakness were common symptoms preceding death. Hepatic pathology included discoloration of the liver, extensive necrosis of the parenchyma, and diffuse distribution of necrotic hepatocytes (62). This pathology varied from the well-circumscribed necrotic foci characteristic of RVFV, a bunyavirus for which WSLV is commonly misdiagnosed in ruminants (141). This group later investigated WSLV infection in adult sheep. The only clinical symptom was moderate fever, which was observed in 26 out of 33 animals. Further, microscopic liver lesions composed a milder phenotype compared with the necrosis and hepatocyte morphological change observed in newborn lambs. Ultimately, adult sheep were less susceptible to WSLV infection compared with newborn lambs, and WSLV caused less hepatic damage compared with RVFV (63).

Experimental inoculation of pregnant ewes at one-third gestation revealed that WSLV crossed the maternal–fetal interface and was neuroinvasive in fetuses. Ewes were viremic 1–5 dpi, yet viral RNA was not detected in maternal liver, spleen, nor placental-draining lymph nodes. WSLV was detected in a variety of placental and fetal tissue, and strong staining of WSLV antigen occurred throughout the fetal brain in neurons, glial cells, and neural progenitors (142). While human placental explants had previously been shown to support ZIKV replication, they did not support WSLV replication (143).

### Goats

WSLV inoculation of West African dwarf goats caused 100% mortality. Clinical symptoms included weight loss, dehydration, diarrhea, and lymphocytopenia (144). In red Sokoto goats, WSLV caused 50% mortality. Fever coincided with viremia, which began 24–72 hpi and lasted 3–4 days. Tissues were collected from goats that succumbed to lethal infection, and WSLV RNA was detected in the spleen, liver, kidney, brain, lungs, adrenal glands, and mesenteric lymph nodes. Neutralizing antibodies were detected in the surviving animals (145).

### Horses

Albeit rare, neurological symptoms have been observed in wild horses infected with WSLV and MVEV (99, 146). Experimental MVEV infection in 9-month-old foals resulted in minimal symptoms. Viremia occurred in 5 out of 11 horses accompanied by transient fever and reduced appetite. All horses developed hemagglutination-inhibiting antibodies and were ultimately deemed unlikely amplifying hosts (147). Experimental infection of horses with WSLV has not yet been accomplished. Survey studies of USUV in wild horses have been performed in European countries, including Croatia, Poland, Israel, France, and Spain but inoculation of horses in a laboratory setting has yet to be done (53, 148–151).

### Non-human primates

The official amplifying host of SPOV is unknown but is speculated to be non-human primates (NHPs). One group investigated SPOV infection in two macaque species. While rhesus macaques were permissive, cynomolgus macaques restricted SPOV infection; however, SPOV efficiently replicated in primary skin fibroblasts isolated from both species. Additional experiments investigated the protective immunity provided by SPOV or ZIKV infection in heterologous challenges. SPOV-exposed rhesus macaques were susceptible to subsequent ZIKV challenge; however, ZIKV-immune macaques were protected against subsequent SPOV challenge (40). NHP experiments have not been performed for any other aforementioned emerging flavivirus.

## CONCLUSIONS

### Species differences likely contribute to variation in flavivirus susceptibility

Whether a flavivirus replicates and progresses to mild or systemic infection in a particular animal likely depends on virus-specific mechanisms of immune antagonism and host-specific restriction factors. For example, ZIKV NS5 binds and degrades human STAT2, thereby antagonizing the innate immune response; however, it does not interact with mouse STAT2 nor cause disease in mice. The emerging mosquito-borne flaviviruses described above demonstrate lethality in certain animal species but are generally overcome by immunocompetent human hosts. USUV, ILHV, ROCV, MVEV, and ALFV all utilize avian amplifying hosts that have simpler immune systems compared to humans and mice. Birds only have one type of polymorphonuclear leukocyte, yet they have nucleated thrombocytes that are involved in innate immunity. Additionally, birds have reduced antibody diversity due to a simplified process of immunoglobulin gene rearrangement (152, 153). WSLV mainly affects ruminant animals, which have a strikingly high proportion of gamma/delta (γδ) T cells compared to humans and mice (154). Fetal and newborn lambs are most vulnerable to lethal infection, which might be explained by an immaturity of the immune system and permissivity of fetal tissues that is lost with further development. While there are major species differences, subtle evolutionary differences can also have large effects, as seen between human and mouse STAT2 in ZIKV infection. Further studies are required to uncover these nuances.

### Studying emerging flaviviruses is a valuable preventative measure

Flaviviruses continue to cause millions of infections each year with very few effective vaccines and therapeutics available. Neglected flaviviruses have been detected in a variety of animal species, some of which are highly lethal in certain hosts, but have yet to cause extensive human infection. As a result, their research is lowly prioritized. With increasing global temperatures, further expansion of human agriculture, and continued urbanization, regions affected by mosquito-borne viruses are projected to grow (155, 156). The high mutation rate of flaviviruses may lead to the inception of strains with increased virulence for mammalian hosts in which case prior knowledge of these viruses would facilitate reactive public health efforts. Many useful insights can be gained from studying these viruses at present, such as discovery of their cellular receptors and restriction factors. Understanding the shared and unique mechanisms employed by each virus will help explain their individual host and cell tropisms and potential for zoonotic transmission.
